# Mobilizing IDEAS in the Scottish Referendum: Predicting voting intention and well‐being with the Identity‐Deprivation‐Efficacy‐Action‐Subjective well‐being model

**DOI:** 10.1111/bjso.12355

**Published:** 2019-11-19

**Authors:** Dominic Abrams, Giovanni A. Travaglino, Peter R. Grant, Anne Templeton, Mark Bennett, Fanny Lalot

**Affiliations:** ^1^ University of Kent Canterbury UK; ^2^ The Chinese University of Hong Kong, Shenzhen China; ^3^ University of Saskatchewan Saskatoon Saskatchewan Canada; ^4^ Edinburgh University UK; ^5^ University of Dundee UK

**Keywords:** Relative Deprivation, Nationalism, Social Identity, Voting, Well‐Being

## Abstract

In the month approaching the 2014 Scottish Independence referendum, we tested the Identity‐Deprivation‐Efficacy‐Action‐Subjective Well‐Being model using an electorally representative survey of Scottish adults (*N* = 1,156) to predict voting for independence and subjective well‐being. Based on social identity theory, we hypothesized for voting intention that the effects of collective relative deprivation, group identification, and collective efficacy, but not personal relative deprivation (PRD), should be fully mediated by social change ideology. Well‐being was predicted to be associated with PRD (negatively) and group identification (positively and, indirectly, negatively). Unaffected by demographic variables and differences in political interest, nested structural equation model tests supported the model, accounting for 82% of the variance in voting intention and 31% of the variance in subjective well‐being. However, effects involving efficacy depended on its temporal framing. We consider different ways that social identification can simultaneously enhance and diminish well‐being and we discuss ramifications of the model for collective mobilization and separatist nationalism. Findings also suggest new directions for research on social identity, collective efficacy, and collective action.

Following a 1997 referendum, the devolved Scottish parliament, convened in 1999, reflected a gradually accelerating trend of support for the Scottish National Party (SNP), from 10% in 1979 to 37% in 1997 (McCrone, 2004). Yet control over many of Scotland’s laws, its defence, and tax‐raising power remained in the hands of the UK parliament in London, maintaining a stable power structure. Support for the SNP was fuelled by growing antagonism towards the political and policy agendas imposed by a rightwing (Conservative Party) government majority in the UK parliament. The SNP, emphasizing the attributes and achievements of the Scottish people in the past, appealing strongly to people’s identity as Scottish, and emphasizing the current economic self‐sufficiency of Scotland, argued that Scotland should become a sovereign nation (cf. McCrone, 2004; Reicher & Hopkins, 2001). By 2012, in response to continued public pressure the UK parliament agreed to hold a referendum on full Scottish independence in 2014. Of the 84.6% of the Scottish electorate (3,623,344 people) who voted, 44.7% voting Yes to independence (BBC, 2014). This context provided a societal test case through which to examine social psychological processes in collective mobilization to achieve social change.

Political separatism has not featured extensively in psychological research on intergroup relations (see Abrams & Grant, 2012, p. 675; Becker & Tausch, 2015; Livingstone, Manstead, Spears, & Bowen, 2011; van Zomeren, Postmes, & Spears, 2008). Yet it can be an important goal for radicalized disadvantaged groups in society (Sweetman, Leach, Spears, Pratto, & Saab, 2013). We use an electorally representative sample of Scottish voters immediately prior to the referendum to test the social identity–relative deprivation–efficacy (SIRDE) model (Abrams & Grant, 2012; Grant, Abrams, Robertson & Garay, 2015; Grant, Bennett & Abrams, 2017). We then extend previous conceptualizations in a more comprehensive Identity‐Deprivation‐Efficacy‐Action‐Subjective Well‐Being (IDEAS) model. This builds on recent theoretical advances in the field of intergroup relations that aim to combine ideas from relative deprivation theory, social identity theory, and resource mobilization theory to explain collective actions taken by members of disadvantaged groups who feel that their group is being treated unfairly (e.g., Becker & Tausch, 2015; Sturmer & Simon, 2004; van Zomeren, Leach, & Spears, 2012; van Zomeren, *et al.*, 2008). The IDEAS model extends this to address and link both the social and personal identity implications of relative deprivation. Below we review intersecting lines of research that underpin the hypotheses tested in the model (depicted in Figure 1).

**Figure 1:**
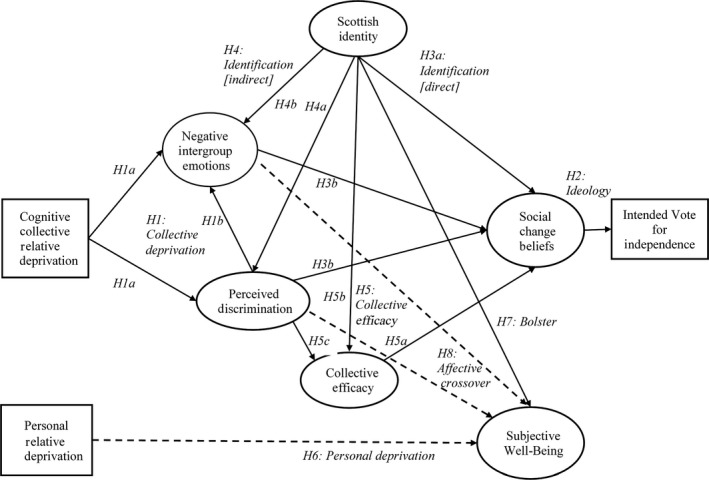
Schematic Depiction of the Identity‐Deprivation‐Efficacy‐Action‐Subjective Well‐Being (IDEAS) model, predicting voting intention and subjective well‐being. Dashed lines indicate relationships that are hypothesized to be negative.

## The Social Identity–Relative Deprivation (SIRD) and Efficacy (SIRDE) models

### Relative deprivation theory

Relative deprivation theory proposes distinct effects of perceived deprivation at the personal and group levels of analysis (Olson *et al.*, 1986; Runciman, 1966; Smith, Pettigrew, Pippin, & Bialosiewicz, 2012; Walker & Pettigrew, 1984). Personal relative deprivation (PRD) arises when a person feels unjustly deprived relative to other individuals in their own reference group. PRD is predicted to result in lower life satisfaction (Crosby, 1976), a point we return to later. In contrast, collective relative deprivation (CRD) results from an intergroup comparison where one’s ingroup as a whole appears to be unjustly deprived relative to a relevant outgroup.

Collective relative deprivation has cognitive and affective components. The cognitive component (cogCRD) is the belief that the ingroup is disadvantaged relative to a relevant comparison group. In Scotland, the cognitive component involves the perception that people working in England are better paid and have better job prospects than those in Scotland. The affective component (affCRD) is the perception that the ingroup’s disadvantage is unfair together with the emotions of anger and frustration at this injustice. Smith *et al.*’s (2012) meta‐analysis of the relative deprivation literature showed that, to date, researchers have measured either one or the other of these aspects of affCRD and that both are stronger predictors of militant attitudes and collective protest actions, their model predicting that affCRD should mediate the effect of cogCRD. Importantly, Grant (2008) has used intergroup emotions theory (Devos, Silver, Mackie, & Smith, 2002; Iyer & Leach, 2008; Smith, 1993) to argue that affCRD is part of a coping response in which discrimination by an outgroup is perceived to be the cause of the ingroup’s disadvantage and that this attribution leads to anger and frustration, a specific emotional response. This perspective is echoed in a recently developed process model of intergroup emotions in which it is argued that ‘appraisals of group‐based unfairness and outgroup accountability underlie the experience of group‐based anger’ (Goldenberg, Halperin, van Zomeren, & Gross, 2016, p. 134). That is, the emotions of anger and frustration energize involvement in collective action to counter a perceived injustice (see also Becker & Tausch, 2015; van Zomeren, Leach, & Spears, 2012).

*Hypothesis 1a* is, therefore, that the more the Scots perceive that Scottish people are disadvantaged relative to the English (cognitive CRD), the more that they will believe that Scottish people are being discriminated against collectively and the more they will feel the intergroup emotions of anger and frustration – the two elements of affective CRD. *Hypothesis 1b* is that the two elements of affCRD are related such that greater perceived discrimination is likely to increase levels of anger and frustration.

### Social identity theory

Social identity theory (Tajfel, 1978; Tajfel & Turner, 1979) holds that social comparisons between people’s own groups (ingroup) and other groups (outgroups) are central to the meaning and value of their group memberships. When individuals identify with a group, their group membership becomes part of their collective self‐concept. However, when members of a low‐status group compare their group to the dominant group in society, the implications for their self‐concept are often negative. The result is that low‐status group members use various strategies to protect and enhance their group identity motivated by their drive to maintain a positive self‐concept (see Abrams, 2015; Hogg & Abrams, 1988). They can, for example, join another group, compare their group to another less prestigious group, or make an intergroup comparison using a different dimension (Tajfel & Turner, 1979). Further, if they feel that their group’s low status is illegitimate, they might engage in collective protest actions to right this perceived injustice and improve their group’s status directly. It is under these circumstances that social identity theory and relative deprivation theory converge. Indeed, Tajfel developed social identity theory, in part, to ‘articulate some of the social psychological processes which are responsible for the genesis and functioning of relative deprivation’ (Tajfel, 1978, p. 67).

van Zomeren, Postmes, and Spears (2008) meta‐analysed a large number of studies predicting collective actions taken by members of disadvantaged groups in order to improve their group’s illegitimately low status. They deduced and meta‐analytically supported an integrated social identity model of collective action (SIMCA). Specifically, a relevant subset of these studies showed that group identity predicted involvement in collective protest actions both directly and through affective injustice, a set of variables that measured unfairness or the emotion of anger arising from an injustice.

Abrams and Grant (2012; Abrams, 1990) have argued that people who feel strongly bonded to their group are most likely to care about the fairness of their group’s status. If they perceive their group’s status to be illegitimately low, they will therefore be more likely to work together to change the status quo, rather than abandoning the group. This is because group members with a strong group identity are most likely to experience socialization by other members (Levine & Moreland, 1994), develop meaningful social relationships within the group (Deaux, Reid, Mizrahi, & Ethier, 1995), and establish a bedrock of shared narratives or elaborated accounts and explanations for intergroup differences. Hence, a strong group identification creates a psychological barrier to considering leaving a group even when its status is in jeopardy (Abrams, Hinkle & Tomlins, 1999; Ellemers *et al.*, 1997). Stronger identification is likely to stimulate both greater outrage at that group’s illegitimate and unfair low status (Kawakami & Dion, 1995) and greater attention to addressing the causes of the injustice.

However, social change within a society can only be achieved if future stability of the current social system is in question. Hence, the SIT hypothesis is that members of disadvantaged groups are most likely to take part in collective protest actions if their group’s status is considered to be illegitimately low and if there is a realistic possibility that such actions might achieve positive change (the social system is not permanent).

Grant *et al. *(2017) have noted that most intergroup relations researchers have studied collective protest actions under circumstances where disadvantaged group members believe that their group’s status in society can be improved through their protest actions (see Smith *et al. *(2012) and van Zomeren *et al. *(2008) for comprehensive reviews of this literature) and where group members are protesting to achieve greater *social inclusion* for their group. In contrast, very little research has examined when group members consider the more radical option of separatism (*social exclusion*) in order to establish their group’s own autonomous society. Theoretically, this refers to the central question of how a strong and positive group identity is sustained in the context of long‐standing (hitherto stable) disadvantage. When a group has illegitimately low social status which, over time, has been very hard or impossible to change within the existing, stable social structure, its members face an increasingly pressing dilemma. Should the disadvantaged group members act collectively to improve their group’s historical status in society in the face of intractable obstacles, or should they advocate for more collective autonomy which would allow them to effect social change more easily?

Abrams and Grant (2012) argue that the latter, more risky option, is dependent on the presence of the development of what Tajfel and Turner (1979) refer to as a social change belief structure, that is, an ideology that an improvement in the group’s situation requires a realignment of power through structural (and in the present case, political) separation. More specifically, the SIRD model proposes that when existing power structures are perceived to present a stable (enduring) but illegitimate obstacle to overcoming collective disadvantage, affective CRD and social identification together provide the foundation for the development of social change beliefs or ideology, which are then the proximal predictor of intentions to support radical change to the existing social structure, in the form of separatism.

Thus, the *ideology hypothesis* (*Hypothesis 2*) is that social change beliefs should be the strongest predictor and proximal mediator of intentions to vote for independence. Specifically, the relationships between voting intention and identification and relative deprivation should all be mediated by social change beliefs. The proximal predictors of social change beliefs are social identification and affective CRD. Thus, for these direct paths the *social identification hypothesis* (*Hypothesis 3a*) is that people who identify more strongly with their group are more likely to adopt a separatist social change belief structure. Further, the *relative deprivation paths *(*originating with Hypothesis 1*) are those from both higher levels of perceived discrimination and more negative intergroup emotions to social change beliefs (*Hypotheses 3b*)*.*

We also proposed an *indirect* effect of identification (*Hypothesis 4*) because those who identify more strongly with Scotland are more likely to perceive its disadvantage as discriminatory (*Hypothesis 4a*) and then feel angrier and more frustrated about it (*Hypothesis 4b*)*.* These indirect paths are implied by both the SIMCA model, which postulates an indirect route from identity to affective injustice (van Zomeren *et al.*, 2008), and also by the dynamic dual pathway model (van Zomeren, Leach, & Spears, 2012).

### Perceived collective efficacy

van Zomeren, Postmes, and Spears’ (2008) meta‐analysis showed that group members are more likely to engage in protest action if they believe that their group can effect positive change. SIMCA, which was derived from the results of this meta‐analysis, postulates that identity influences collective efficacy as well as affective injustices and that all three motivate collective protest actions directly. Research in Chile also indicates that the influence of perceptions of stability and group identification on the perceived legitimacy of social action are mediated by collective efficacy (Jiménez‐Moya, Miranda, Drury, Saavedra, & González, 2019).

Collective efficacy was initially part of a resource mobilization theory perspective on participation in social protests (Klandermans, 1984, 1997, 2004). Later it was specified as a pathway of two influential dual pathway models (see Abrams & Randsley de Moura, 2002). Sturmer and Simon (2004) argued that collective efficacy and group identity constituted the two pathways. Later, van Zomeren *et al. *(2013) argued that collective efficacy and group‐based anger constituted problem‐focused and emotion‐focused approach coping, respectively, which together lead group members to reappraisal their group’s unfair disadvantage and motivate them to participate in collective protest actions. In a prospective study of skilled Canadian immigrants protesting to achieve greater inclusion into Canadian society, Grant *et al. *(2015) further developed the SIRD model to include a collective efficacy component (SIRDE: the social identity–relative deprivation–efficacy model) and found that those who felt more collective efficacy were more likely to engage in normative protest actions.

Subsequently, Grant *et al. *(2017) tested the SIRDE model with a sample of 537 High School students from the Scottish city of Dundee, shortly after the Scottish Independence Referendum. Grant *et al. *(2017) argued that members of a group with an illegitimately low social status would be particularly likely to support separatism if they believe that they lack the ability to effect positive social change within the existing social structure (low collective efficacy). Therefore, they hypothesized and found that those respondents who *lacked* collective efficacy were more likely to hold separatist social change beliefs and these beliefs, in turn, were strongly related to their having voted for independence – a form of social exclusion.

Grant *et al.*’s (2017) theoretical rationale for efficacy has parallels with that proposed by Becker and Tausch (2015) who argued that a lack of perceived collective efficacy and the emotion of contempt motivate engagement in extreme and violent collective protest actions. Because repeated, unsuccessful attempts at reconciliation engender feelings of powerlessness among members of disadvantaged groups, they act destructively as an expression of their contempt for a society which is perpetuating their group’s disadvantage. The SIRDE model instead focuses on the emotion of anger, proposing that it motivates support for separatism if attaining independence from the intergroup relationship responsible for ingroup disadvantage is a viable political option. In Scotland, this is certainly the case as independence is advocated by the political party in power in the Scottish parliament. Therefore, those supporting separatism are likely to do so both because the existing political structure makes them feel powerless to effect positive social change for the Scottish people within the United Kingdom, and because they believe that Scotland’s independence is the means to effect such change.

Grant *et al.*’s (2017) argument on efficacy is intriguing but their study provided only modest evidence in support of the counterintuitive lack of efficacy–social change belief hypothesis. This is because voters who viewed Scotland as having become more disempowered due to the referendum result might have become especially motivated to work towards independence. It is not known whether, prior to the referendum, intentions to vote to leave would have been motivated more by higher collective efficacy, as predicted by SIMCA, or lower collective efficacy, as proposed by Grant *et al. *(2017).

We, therefore, hypothesize that higher collective efficacy should be associated with stronger separatist social change beliefs (*Hypothesis 5a*) and that stronger identification should be associated with greater collective efficacy (*Hypothesis 5b*). Because Grant *et al. *(2017) hypothesized (and found) that a *lack* of collective efficacy would be associated with separatist social change beliefs, we consider hypothesis 5a to be tentative. Although prior evidence is mixed (e.g., Grant *et al*, 2015, 2017), we also speculate that stronger perceived discrimination should predict greater efficacy because group members who perceive more discrimination may conclude that collective action may resolve the situation (*Hypothesis 5c*).

## The present study – the IDEAS model

The two previous studies of Scottish nationalism that tested the SIRD(E) models involved only young people with limited political experience or who had never voted. One (Abrams & Grant, 2012) was conducted 30 years ago and the other (Grant *et al.*, 2017) was conducted on a school‐based sample after the results of the 2014 referendum were already known. Both studies only involved young people from a single region within Scotland. Thus, the generalizability of the findings and the theory to the actual population of adult voters is unknown. Both, however, point to a key role for social change beliefs, a construct that has been largely overlooked in previous social identity research.

A further question is whether or how it is possible to reconcile the efficacy findings from Grant *et al.*’s (2017) study of voting, with those predicted by the SIMCA in relation to collective action (Van Zomeren *et al.*, 2008). An important task for the present research is to understand whether Grant *et al.*’s (2017) finding is anomalous or whether it might tell us something interesting about when, how and why collective efficacy relates to collective action.

The present research addresses these issues and employs the fuller set of measures used by Grant *et al. *(2017), which include both collective efficacy and a more differentiated measure of relative deprivation and emotion responses than those used by Abrams and Grant (2012). Independence referenda represent unique decision points at which well‐specified structural/legal change may follow. Unlike general or routine elections, they have no behavioural precedent and are not confounded with popularity of particular candidates, so voting cannot be based on habits, traditions, or loyalty to particular representatives. Because the present research sampled the entire electorate in Scotland immediately prior to the referendum, we can test theory more robustly, including its predictive validity in the light of the referendum result.

### Subjective well‐being

The IDEAS model also considers a second outcome associated with relative deprivation, namely subjective well‐being. Crosby (1976) indicated that PRD should affect subjective well‐being but no theories have explicated the way that relative deprivation and social identification might have combinatory effects on well‐being. We propose that there is likely to be an affective crossover between affCRD and subjective well‐being, that is, people who feel discriminated against or angry and frustrated about their collective situation may also feel less happy and satisfied with their life in general. Thus, both personal and CRD can have separate negative impact on well‐being. This is consistent with Schmitt *et al.*’s (2014) meta‐analytic finding that both types of discrimination are associated with lower well‐being.

Second, stronger group identification should have counteracting, effects on well‐being. By increasing sensitivity to intergroup discrimination, identification should contribute to more negative intergroup emotions which engenders *lower* well‐being. However, a positive group identity should also *bolster* well‐being directly by providing meaning and social solidarity. Although a recent meta‐analysis showed that the evidence for the identification – well‐being relationship is very mixed (Schmitt, Branscombe, Postmes, & Garcia, 2014), and perhaps context dependent (Sellers & Shelton, 2003), our reasoning is consistent both with the rejection–identification model (Branscombe *et al.*, 1999) and with the ‘Social Cure’ model proposed by Jetten *et al. *(2012). To our knowledge, the dynamics between identity and well‐being have yet to be integrated into a model of social change and collective action. The IDEAS model therefore sets out the ways that CRD can have both negative and positive effects on well‐being through its cognitive and affective routes in combination with social identity.

The *personal deprivation* hypothesis (*Hypothesis 6*) is that higher PRD should be associated with lower subjective well‐being (see also Schmitt *et al.*, 2014).

Based on theorizing that there should be psychologically beneficial effects of social identification (Jetten *et al.*, 2012), we also propose a *bolster* hypothesis such that stronger group identification should be associated with greater subjective well‐being (*Hypothesis 7*).

Further, in line with Grant *et al. *(2017) and much other evidence (Pascoe & Smart Richman, 2009; Schmitt, Branscombe, Postmes, & Garcia, 2014), we propose an *affective crosso*ver hypothesis (*Hypothesis 8*), whereby perceptions of discrimination and the negative intergroup emotions of anger and frustration (as components of affCRD) are likely to be associated with lower subjective well‐being. Consequently, social identification could have a positive effect on well‐being, but also indirect negative effects via the experience of discrimination and the negative affect created by the unjust treatment of the disadvantaged ingroup (affective CRD). Note that, whereas the rejection–identification model assumes that perceptions of discrimination lead to identification, the model we derived from social identity theory and relative deprivation theory holds that identification is likely to heighten sensitivity to discrimination, resulting in a negative indirect effect on well‐being.

Finally, we do not expect collective efficacy to influence subjective well‐being, in part because they are different levels of the self‐concept (collective and personal, respectively), and for similar reasons, there should not be significant paths from personal RD to either social change beliefs or voting intention. Due to space limitations, these and other hypothesized ‘zero’ paths are considered in more detail in the Supplemental Materials (Figure S3).

## Method

### Sampling and procedure

The methodology and measures were approved by the University’s Psychology Ethics Panel, and all participants gave full consent and had the right to withdraw their (anonymized) data via a personal code. We recruited a broadly representative sample of the Scottish population eligible to vote in the Independence Referendum (ONS, 2018) using Qualtrics Panels. Because of the narrow window for data collection, we set quotas for representation across different regions within Scotland, and a full age distribution for respondents. The sample size was set to be 1,000. The survey was launched on 3 September 2014 and concluded on 14 September 2014, 4 days prior to the referendum itself. Towards the close of the survey, we slightly oversampled to ensure that sufficient numbers of young voters were included to facilitate comparisons with previous studies. The oversampling yielded 1,177 respondents; 21 were not eligible to vote and 144 did not intend to vote and therefore were discarded, leaving a final *N* of 1,012. Analyses commenced following termination of data collection. Participants were paid £5.

Details of the demographic characteristics of the sample are as follows: 51% identified as male, 49% as female; 26.6% were under 21 years of age, 23.3% between 21 and 40, 26.4% were 41–60, and 23.8% were over the age of 60; 40.7% were single, 39.4% married, 10.4% living with another, the remainder married, separated, or divorced; 73.3% were in family‐owned houses, 17.2% were in rented accommodation, and 9.5% did not know. As markers of socioeconomic status, 52.9% owned a car or a van, 29.9% owned more than one car or van, and 17.2% owned neither; 33.7% had at least an undergraduate degree; 38.3% were religious. Intended voting was 46.3% in favour of independence (as compared with the 44.7% of votes cast in the Referendum itself).

### Measures

The measures largely matched those in Grant *et al. *(2017) and were adapted from previous studies by Abrams and Grant (2012) and Grant (2008; Grant *et al.*, 2015). Items and scaling are shown in Table 1. The majority of items used 5‐point scales with acceptable or good reliability coefficients. Additional items measured variables that are not central to the theory tested in this paper. Where possible, constructs were operationalized as latent variables in SEM. For ease of interpretation, we also report the alpha reliability coefficients for composite scores which were created by averaging the items in each scale. The composite scores were used to calculate the correlations among variables in Table 2. Multi‐item measures were as follows: Cognitive CRD (three items), Affective CRD comprising a Perceived Discrimination measure (four items), and a Negative Intergroup Emotions measure (six items); Collective Efficacy (three items), Separatist Social Change Beliefs (three items), and Subjective Well‐Being (two items, see Diener, 1994; Swift, Vauclair, Abrams *et al.*, 2014). Single item measures were used for Voting Intention, Political Interest, and PRD.

**Table 1:** Constructs and measures

Construct	Reliability	Question	Scale
Cognitive Relative Deprivation	.77	Compared to the standard of living for English people living in England, the standard of living is… Compared to job opportunities for English people living in England, job opportunities for Scottish people are… Compared to the wages of English people living in England, wages for Scottish people living in Scotland are…	1: Much worse 5: Much better
Scottish Identification	.88	I feel Scottish I am proud to be Scottish Being Scottish is important to me I have strong ties with other Scottish people My views about Scotland are shared by other Scottish people	1: Not at all 7: Extremely
Emotions	.91	When you compare the standard of living of Scottish people living in Scotland with that of English people When you compare the job opportunities of Scottish people living in Scotland with that of English people When you compare the wages of Scottish people living in Scotland with that of English people	*(How do you feel? [Angry’/‘Frustrated’ averaged]* 1: Not at all 4: Very extremely
Discrimination	.85	English employers think that a Scottish education is inferior to an English education When Scottish people are in England, they face discrimination because of their accent When Scottish people are in England, they face discrimination when they look for employment English people do not appreciate Scottish culture and traditions	1: Strongly agree 5: Strongly disagree
Collective efficacy (a priori model)	.67	Nowadays, Scottish people are the ones in control of Scotland’s future as a country Because of their shared goals, Scottish people are the ones who have the most influence over the direction taken by Scotland as a country Together, Scottish people are the ones who decide Scotland’s future	1: Strongly disagree 5: Strongly agree
Collective efficacy (final model)	.63	Because of their shared goals, Scottish people are the ones who have the most influence over the direction taken by Scotland as a country Together, Scottish people are the ones who decide Scotland’s future	1: Strongly disagree 5: Strongly agree
Ideology	.90	Scotland could get along quite well without the rest of Britain It is important for Scotland to have control and manage its own resources People in Scotland will only get a fair deal if Scotland is an independent country	1: Strongly disagree 5: strongly agree
Personal Relative Deprivation	N/A	Comparing your own standard of living and how much money you have with that of other Scottish people your age, do you think you have….	1: Much less 5: Much more (reverse coded for analysis)
Subjective Well‐Being	.89	All things considered, how satisfied are you with your life as a whole nowadays? Taking all things together, how happy would you say you are?	1: Very dissatisfied/unhappy 5: Very satisfied/happy
Political Interest	N/A	Generally, how interested are you in politics?	1: Not at all 5: Very interested
Vote Separatist	N/A	If the referendum on Scottish independence were held tomorrow, would you vote YES or NO to the referendum question? Should Scotland be an independent country?	1: Yes 2: No

Spearman–Brown coefficient is used rather than Cronbach’s alpha for assessing reliability of the 2‐item measures.

**Table 2:** Means, standard deviations, and correlations among the variables (*N* = 1,012)

Dependent variable	1.	2.	3.	4.	5.	6.	7.	8.	9.	10.	11.
1. Cognitive CRD	1										
2. Negative Collective Emotions	.49***	1									
3. Discrimination	.25***	.56***	1								
4. Scottish Identification	.23***	.26***	.28***	1							
5. Collective Efficacy (1,2, 3)	−.10**	.08**	.01	.10**	1						
6. Collective Efficacy (2 and 3)	−.002	.17***	.12***	.17***		1					
7. Collective Efficacy (item 1)	−.21***	−.08*	−.16***	−.06			1				
8. Social Change Beliefs (separatism)	.33***	.54***	.57***	.39***	.09**	.25***	−.21***	1			
9. Vote for Independence	.33***	.47***	.50***	.36***	.06	22***	−.22***	.87***	1		
10. Personal RD	.28***	.17***	.10**	.08	−.01	.02	−.07	.15***	.15***	1	
11. Subjective Well‐Being	−.33***	−.29***	−.22***	.004	.06	−.003	.14***	−.23***	.23***	−.50***	1
Mean (*SD*)	3.30 (0.64)	1.71 (0.79)	2.88 (0.92)	5.78 (1.02)	3.29 (0.88)	3.48 (0.95)	2.92 (1.17)	3.29 (1.32)	0.46 (0.50)	2.87 (0.92)	3.65 (0.86)

*p < .01; **p = .001; ***p < .001, two‐tailed.

## Results

### Analytic strategy

**Figure 2:**
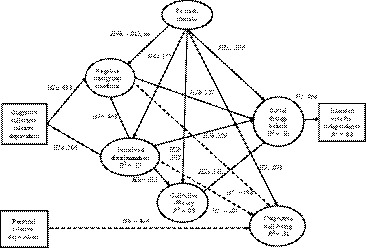
Coefficients for Model 2c. All paths are significant at *p* < .01 unless otherwise indicated. Negative paths are dashed lines.

We used the Lavaan SEM package in R to test a series of models, using the Yuan‐Bentler (Y‐B)χ^2^ robust maximum‐likelihood estimator with standardized coefficients (see Figure 2 and Table S3). Given the large sample size, we used *p* < .01 as the criterion for deciding whether a path coefficient or correlation was meaningfully different from zero. The three exogenous variables were initially allowed to covary to account for any shared variance associated with unknown common variables. As the CogCRD‐ PRD and CogCRD‐ identification correlations were significant, these were retained in the series of model tests reported below.

Table 2 provides the means, standard deviations, and correlations among individual items and composite scores representing the latent variables that were later used in the structural equation analyses.

#### Model 1

The first model tested the paths hypothesized to be significant in the IDEAS model (Figure 1). This had acceptable fit to the data (see Hair *et al.*, 2010), RMSEA = .059, SRMR = .064, CFI = .939, χ^2^(259) = 1,175.84 = 37, *p* < .001, and all but one of the predicted paths were significant. We consider the SIRDE hypotheses first: Consistent with the *ideology hypothesis* (H2), separatist social change beliefs was a very substantial significant predictor of voting intention (β = .90, *p* < .001). All relationships between other variables and voting intention were fully mediated by separatist social change beliefs. Adding direct paths from identity, intergroup emotions, discrimination, and collective efficacy to voting intentions did not improve the model (nor were any of these paths suggested by modification indices). Together, other variables in the model accounted for a substantial portion of the variance in separatist social change beliefs (*R* ^2^ = .50, *p* < .001).

Consistent with *the social identification hypothesis* (H3a), identification with Scotland significantly predicted separatist social change beliefs (β = .20, *p* < .001), and there was a significant indirect effect of social identification on voting intention (β = .33, *p* < .001).

Consistent with CRD *hypothesis* (H1a), respondents who more strongly perceived that Scottish people are disadvantaged relative to the English (cognitive CRD), were more likely to believe that Scottish people were being discriminated against (perceived discrimination (β = .28, *p* < .001) and felt angrier and more frustrated (negative collective emotions) (β = .43, *p* < .001). Consistent with *CRD hypothesis* (H1b), perceived discrimination and negative intergroup emotions were positively related to one another (β = .50, *p* < .001). More importantly, supporting the *relative deprivation hypothesis* (H3b), stronger separatist social change beliefs were reported by respondents who perceived higher discrimination (β = .40, *p* < .001) and who felt more negative intergroup emotions (β = .29, *p* < .001). Moreover, the indirect effect of perceived discrimination on ideology via collective efficacy was non‐significant, (β = .005, *p* = .098), as was the indirect effect of negative collective emotions to ideology via collective efficacy (β = .006, *p* = .077).

In line with the predicted indirect effects (*Hypothesis 4*), perceived discrimination was positively predicted by social identification with Scotland (*H4a*; β = .17, *p* < .001), and there was a significant indirect effect of social identification on negative intergroup emotions via perceived discrimination; β = .144, *p* < .001). However, the path from identification to negative intergroup emotions of anger and frustration was non‐significant (*H4b*; β = −.024, *p* = .387, *ns*).

For the *collective efficacy hypotheses,* there was no support for *hypothesis 5a* that higher collective efficacy should be associated with stronger separatist social change beliefs (β = .03, *p* = .251). Nor was there support for *hypothesis 5b*, as the relationship between perceived discrimination and greater collective efficacy was not significant (β = .05, *p* = .224). However, there was support for *hypothesis 5c* shown by a significant positive relationship between higher Scottish identification and greater collective efficacy (β = .11, *p* = .005). We return to this set of relationships in our *a posteriori* analyses.

Turning to the effects on well‐being, as expected from the IDEAS model, the predictors accounted for a significant proportion of variance in subjective well‐being (*R* ^2^ = .31, *p* < .001). Supporting the *personal deprivation hypothesis* (H6), higher personal deprivation was associated with lower subjective well‐being (β = −.48, *p* < .001).

We also found support for the *bolster hypothesis* (H7), because higher identification was associated with more positive well‐being (β = .13, *p* < .001). Finally, consistent with the *affective crossover hypothesis* (H8), we allowed for, and found, a negative relationship between negative intergroup emotions and well‐being (β = −.19, *p* < 001), and perceived discrimination and well‐being (β = −.23, *p* < .001).

### Further analyses

#### Models 2a–2c – collective efficacy and temporality

An explanation for Grant *et al.*’s (2017) finding of a negative relationship between collective efficacy and separatist ideology may be linked to the timing of the studies. Given that Grant *et al.*’s (2017) study of teenagers was conducted after the referendum, this negative association might be because respondents who strongly advocated separatism had concluded that Scotland’s capacity to control its own future had now become diminished. Given the potential importance of this temporal aspect, we considered the temporal implications of each of the three items that measured collective efficacy in the present research (see Table 1).

The first item focused on Scotland’s current efficacy (‘Scottish people are in control’) whereas the second and third items focused more on prospective efficacy (‘influence over the direction’). Despite the fact that the three items correlated positively with one another, we noticed that the first item correlated *negatively* with separatist social change beliefs, whereas the second and third items correlated positively (see Table 2). This suggested that the first item and second and third might statistically suppress one another’s contribution to ideology. Therefore, *Model 2a* used the two prospective efficacy items as the latent variable for collective efficacy while treating the current efficacy item as a covariate. This model resulted in a larger and now significant *positive* path from collective efficacy to separatist ideology (β = .23, *p* < .001; overall model RMSEA = .075, SRMR = .056, CFI = .941, χ^2^(254) = 1,088.962, *p* < .001).

Conversely, in *Model 2b* we used the first (current efficacy) item as a manifest predictor and treated the two prospective efficacy items as covariates. We found a significant *negative* path from efficacy to ideology (β = −.18, *p* < .001) but the overall model fit reduced; RMSEA = .065, SRMR = .069, CFI = .921, χ^2^(256) = 1,361.77, *p* < .001.

Finally, *Model 2c* dropped the first efficacy item altogether. Consistent with H5a, this revealed a significant positive path from efficacy to separatist social change beliefs (β = .15, *p* < 001), and there was a slight improvement in the model fit RMSEA = .056, SRMR = .045, CFI = .945, χ^2^(232) = 980.205, *p* < .001. Therefore, we retained Model 2c (see Figure 2), before proceeding to check that the models were not compromised by potentially confounding or nonzero paths (see Supplemental Materials for the nested models and tests of indirect effects).

#### Model 3 – zero paths and demographic variables

Finally, we tested the resilience of the model when all other potential confounds and predicted zero paths were added. Model 3 is a highly conservative model test*,* in which we included age, gender, and car ownership as covariates, accounted for political interest as a direct predictor of voting intention, and added the pathways theoretically specified to be non‐significant as well as the originally hypothesized direct effect between identification and negative intergroup emotions (H4b). Two of the predicted zero paths are particularly important if we are to accept the proposition that PRD should affect subjective well‐being but should not influence collective action. These are pathways from PRD to social change beliefs and from PRD to voting intention. Model 3 confirms that neither of these paths contributes significantly (βs = .04 and .01, respectively), RMSEA = .052, SRMR = .047, CFI = .941, χ^2^(313) = 1,181.294, *p* <.001. The fit of Model 3 is not as good as the IDEAS model (Model 1), or its statistically refined version (Model 2c) which are also more parsimonious as they have fewer parameters. Moreover, Model 2c meets all the criteria for adequate fit (including an SRMSR of <.055, Hair *et al*., 2014).

## Discussion

The present evidence supports the IDEAS model proposition that relative deprivation and social identification have combined influences on collective action and subjective well‐being. Social identity and CRD predict social change beliefs, which predict behavioural intentions. Personal deprivation is associated with lower well‐being. In contrast, social identity has a direct and positive effect, but an indirect negative effect on well‐being through affective CRD – the affective crossover. That is, the respondent’s Scottish identity both predicted well‐being while also affecting well‐being negatively because it was associated with stronger perceptions of discrimination and negative group‐based emotions (see also Table S1). Thus, while being distinct outcomes, collective action and subjective well‐being have common roots in relative deprivation and social identity.

The Scottish Referendum mobilized the highest percentage turnout of eligible voters ever seen in a UK referendum or election (84.6%). It provides one of the best available large‐scale cases of political mobilization. Rather than just being a study of minority action, the present research involved a whole population that was quite evenly divided in its preferences. This allowed a meaningful and very strong real‐world test of whether the IDEAS model could predict the decision either for or against independence. The voting intentions in our sample closely mirrored the votes cast by the population. Research using similar intention measures in the context of the Brexit referendum indicates a very high correlation between stated intentions and actual voting behaviour (Van de Vyver *et al.*, 2018). The strikingly high correspondence between the more abstract social change belief structure and the concrete imminent voting intention in the present study, together with the close match with the actual pattern of voting in the subsequent referendum suggests that the IDEAS model is well suited for predicting action in situations where pivotal collective decisions are centrally relevant to social identity in intergroup contexts.

It is important to state that there was no suggestion from modification indices that voting intention and social change beliefs were part of the same latent factor. In addition, both the content and format of the items measuring social change beliefs differ from the voting intention measure, so we are confident that they are not simply measuring the same thing. Moreover, the relationship between social change beliefs and voting is not always as high (e.g., it is .32 in Abrams & Grant, 2012). It seems likely that the imminence of the opportunity to vote may have strengthened the relationship in the present research.

Supporting the ‘social change’ part of the model, both identification with Scotland and affective CRD predicted voting intentions entirely through separatist social change beliefs (see also Tables S2 and S3). Social identity and relative deprivation provided the motivation and justification for adopting the view that the group’s situation could only be improved by a structural change. In line with the IDEAS model, the higher collective efficacy predicted a greater propensity to act to improve the group’s situation (as predicted by the SIMCA). It is important to note that given a presently stable intergroup structure, this happened only indirectly through separatist social change beliefs.

Van Zomeren *et al.*’s (2008) meta‐analysis provided consistent evidence that relative deprivation (affective injustice) and social identification promote collective action aimed at greater social *inclusion* (rights or voice within the system) for minority ingroups (see Becker & Tausch, 2015 who also make this point). The present theory and research show how relative deprivation and social identification can provide a basis for more radical social change, in the form of collective exit, separating completely from the existing power system, thus achieving collective *exclusion* from that system in order to attain autonomy. In line with Tajfel’s early theorizing and in the light of the very many historical instances of separatist/independence movements (particularly as empires dissolve), it is important that intergroup relations theory should continue to explore this ultimate expression of social change ideology more fully. Indeed, because of the changing political landscapes, the rise of populism, and nationality‐based resistance to population movement across the globe (Abrams & Travaglino, 2018), the questions of why and when people mobilize to gain or maintain freedom from superordinate political and constitutional structures pose an important and significant contemporary challenge for intergroup relations theory.

Grant *et al. *(2017) hypothesized and found that Scottish teenagers who *lacked* collective efficacy were more likely to hold separatist social change beliefs which, in turn, were strongly related to the vote for independence in the recently held Scottish referendum. However, this finding was obtained after the referendum when perceptions of low efficacy may have bolstered separatist ideology. In contrast, the present study took place just before the referendum took place. Despite age differences between the samples, age within the very age diverse present sample did not qualify effects associated with efficacy.

We discovered that respondents’ perceptions of lower current efficacy were associated with stronger social change beliefs, whereas perceptions of higher prospective efficacy (conditional on a successful campaign) were associated with stronger social change beliefs. This suggests that collective efficacy may relate differently to other collective action variables depending on the temporal reference point or the timing of measurement of efficacy relative to pivotal moments of potential change (cf. Klein *et al.*, 2007). Clearly, then, the role of different aspects of efficacy in motivating various type of collective protest actions warrants further research (see also Becker & Tausch, 2015; Saab, Spears, Tausch, & Sasse, 2016; van Zomeren, Saguy, & Schellhaas, 2013).

Counter to hypothesis 4b, no significant direct relationship was obtained between Scottish identification and the intergroup emotions of anger and frustration. This finding was unexpected because the hypothesis was suggested by evidence supporting SIMCA (van Zomeren *et al.*, 2008), and the dual pathway model (van Zomeren *et al.*, 2013), and in a direct test of the SIRDE model (Grant *et al.*, 2017). Although existing evidence does not enable us to provide an explanation for this finding, this result does highlight the value of separating perceptions of injustice (discrimination) from the intergroup emotions of anger and frustration in the model in order to capture their distinct motivational roles.

Social identity theory initially held that social and personal identity corresponded to intergroup and interpersonal phenomena respectively with no necessary connection between the two (see Hogg & Abrams, 1988). The literature on relative deprivation similarly has tended to emphasize that personal and CRD corresponds to different types of response. However, subsequent work argued that people do link their personal and social identities both subjectively and also in the course of regulating their social behaviour. Thus, there is now a greater acceptance that there is sometimes a strong connection between intragroup and intergroup dynamics (e.g., Marques & Páez, 1994), between different aspects of social identity (e.g., Brewer & Gardner, 1996), and the coordination of identity through people’s self‐regulation as a group member (Abrams, 2015). Similarly, it is more clearly recognized that personal outcomes, such as well‐being, health, and leaving the group, are phenomena that can be linked to social identity (Abrams, Ando & Hinkle, 1998; Jetten *et al.*, 2012).

The IDEAS model explicitly accommodates cross‐connections between the personal and social influences. Specifically, while we predicted and found that PRD bears directly on subjective well‐being but not on social change beliefs, we also found that social identity has two types of contrasting effect on well‐being. First, it bolsters well‐being so that stronger group identification is associated with higher subjective well‐being. Second, given the context of collective disadvantage, stronger identification can result in *lower* well‐being through the affective crossover from negative collective emotions and perceptions of discrimination. Thus, stronger identification with a group that is perceived to be suffering may also mean that individual members’ well‐being suffers.

The Elaborated Social Identity Model (Drury & Reicher, 1999) holds that engagement in crowd action can induce positive affect and hence well‐being. Yet this idea has not been extended to collective action that arises in isolation, such as voting. Moreover, as far as we are aware the IDEAS model is the first to have explicitly linked both the collective action and subjective well‐being outcomes to identity and relative deprivation in the same model. Thus, it extends and links both research on collective action, such as the SIMCA model (Van Zomeren *et al.*, 2008), and research on social identity and well‐being, such as the social cure approach (Jetten *et al.*, 2012) and the EISM. In particular, the IDEAS model does not pit predictions of positive and negative effects of social identity against one another (cf. Schmitt *et al.*, 2014), but rather assumes that they can arise in parallel. Our findings, show for example, that the effect of identification on well‐being can arise via perceived discrimination, but not vice versa (in contrast to the rejection–identification model), but we accept that the directionality of this path could be reversed under different circumstances (see Supplementary Materials for details). Thus, even if an overall relationship between social identity and well‐being is small or non‐significant, this might mask the presence of parallel but contrasting effects, which can potentially be quite strong.

### Implications, limitations, and future directions

The counteracting relationships between social identity and well‐being raise the further interesting question of whether social identity could have counteracting effects on mobilizing collective action via well‐being itself. For example, future research could investigate whether there is a threshold at which well‐being becomes so low that it immobilizes collective action. Conversely, it is plausible that a degree of unresolvable personal discontent might be required or sufficient to propel people to examine intergroup causes of their situation, and hence begin to adopt social change beliefs. Two further avenues for investigation are whether present or anticipated stability of the social system does indeed moderate other effects, and whether there is a feedforward from having participated in collective action to subsequent levels of identification and well‐being (see Vestergren *et al.*, 2017).

The present research focused only on ingroup identification. A further interesting question is whether identification at different levels (e.g., Scottish, British, European) may contribute to differing degrees to the motivation to exit from the intergroup relationship, such as is being seen in attitudes to Brexit in the United Kingdom (see Abrams & Travaglino, 2018; Peitz *et al.*, 2018; Van de Vyver *et al.*, 2018). Although identification with Scotland was an important predictor of social change beliefs, it was not a direct predictor of voting intentions (cf. Sindic & Reicher, 2009) and it may be that disidentification with Britain also enables identification with Scotland to gain more traction.

Much research on collective action and protest relies on limited or unique samples such as relatively small samples of students who are asked to focus on a group membership that is made transitorily salient for the purposes of a study, or distinct subgroups of activists. Similarly, the present theory and measurement were derived from prior studies involving smaller, more specific or local populations. In the Scottish Referendum, all people in Scotland were exposed to the arguments for and against independence over many months and almost all were motivated to vote. The model we tested and the processes that we expected to operate should be reflective of the whole population and are not limited to subsets, networks, or opportunity samples. By sampling the voting age population across Scotland, we were able to establish much greater confidence in the generalizability of both empirical and theoretical indications from previous research and the potential wider value of the IDEAS model for other contexts. Although the present data are cross‐sectional, earlier tests of the SIRD model indicated strong stability over 3 years (Abrams & Grant, 2012), and although we cannot be certain of the causality, we know that voting intention was not based on prior voting (no opportunity had existed), and there is no statistical justification for reversing the flow of the remainder of the model. Nonetheless, longitudinal and experimental tests of the IDEAS model would be worth pursuing in future.

Beyond referenda and separatism, the model could be applied to situations in which an intergroup history has provided time and context for group members to develop an ideological stance on social change. The present findings underline the importance of locating and interpreting evidence in its historical context (Abrams & Eller, 2017). We think the model may inform understanding of extremist groups, of political populism, and forms of system rejection (see Jost, 2018; Travaglino *et al.*, 2017), and can potentially accommodate perspectives that have been used to predict collective action, such as system justification theory (Jost, Becker, Osborne, & Badaan, 2017; see also Supplementary Materials). There are also fascinating possibilities for understanding how social change movements may draw on personal suffering to strengthen social change ideology.

## Data availability statement

The data that support the findings of this study are available on request from the corresponding author. The data are not publicly available due to privacy or ethical restrictions.

## Supporting information


**Data S1** Supplementary Materials.
**Table S1** Model fit statistics for different variants of the model tests.
**Table S2** Indirect Effects on Social Change Beliefs and Subjective Well Being.
**Table S3** Indirect Effects on Voting Intention via Social Change Beliefs.
