# Supplementary material for: Mobilizing IDEAS in the Scottish Referendum: Predicting voting intention and well‐being with the Identity‐Deprivation‐Efficacy‐Action‐Subjective well‐being model
Source: Br J Soc Psychol. 2019 Nov 19;59(2):425–46. doi: 10.1111/bjso.12355 (PMC7186818; doi:10.1111/bjso.12355)
Supplement: Supplementary file 1 — Data S1 Supplementary Materials. Table S1 Model fit statistics for different variants of the model tests. Table S2 Indirect Effects on Social Change Beliefs and Subjective Well Being. Table S3 Indirect Effects on Voting Intention via Social Change Beliefs. [file BJSO-59-425-s001.docx]

**Mobilizing IDEAS in the Scottish Referendum: Predicting Voting Intention and Well-Being with the Identity-Deprivation-Efficacy-Action-Subjective Well-Being Model**

**Supplementary Materials**

**Nested Model Tests**

A full description of the fit of different nested models is provided in Table 1 below. Here we discuss the basis for inclusion of particular variables at different steps in the sequence.

**Models 1b and 1c– Political Interest and Age of Respondents.**

People may become motivated to vote simply by their level of interest in politics. Although we assumed that political interest and ideology should not be related, it is conceivable that those who are more engaged also support more radical positions (see also Abrams & Grant, 2012). If this were true, the inclusion of political interest in the model would reduce the explanatory power of social change beliefs. Political interest (*M* = 3.37, *SD* = 1.07) was significantly correlated with a number of other variables: (** *p* < .01, *** *p* < .001: Cognitive CRD -.02; Negative Emotions .09**; Discrimination -.02; Scottish Identity .06; Collective Efficacy item 1 .06; Collective Efficacy items 2 and 3 .09**; Social Change Beliefs .11**; Personal RD -.15**; Subjective Well-Being .13***; Voting Intention .10**. Therefore, it seemed appropriate to test whether it also independently contributed to the prediction of voting intention.

Respondents’ age is also relevant because older people’s greater political experience may result in deeper political commitment. Indeed, we observed some significant or near significant correlations between age and other variables (see Table 2). Age might also explain another aspect of the present findings. The small positive correlation between collective efficacy and social change beliefs (see Table 1) is consistent with a test of the SIRDE model in a different context (Grant et al., 2015), and with much prior research (Van Zomeren et al, 2008). However, it is inconsistent with Grant et al.’s (2017) post referendum finding among teenagers, revealing *negative* relationships between these variables. One explanation could be that collective efficacy plays a different role in younger people’s political intentions, perhaps because of their relative lack of political experience or voting. Another possibility is that low efficacy might encourage social change beliefs in younger people but, as people get older, sustained failure to have achieved change begins to dampen or reverse that motivation. To explore these possibilities, we compared respondents aged under 30 with those aged 31 and over to test whether the correlations between collective efficacy and ideology (social change beliefs) differed in these two subsamples. However, the relationships turned out to be similarly small among those aged 30 and under, *r* = .07, *p* = .159, and those 31 and over, *r* = .09, *p* = .029).

To account for the potential roles of political interest and of age, *Model 1b*, included the path from political interest to voting intention but found no significant effect (β = .02, *p* = .258). *Model 1c*  further added respondents’ age as a covariate but this made no difference to the sizes or significance (*ps* remained < .01) of other paths in the model, and seemed to slightly reduce the fit of the model overall..

**Model 2d - Demographic Variables**

Given that we conducted a cross sectional study it was important to investigate whether relationships among the theoretically specified variables might be attributable to stable longer term contextual factors. To that end we measured relevant socio-demographic variables with a view to controlling for or understanding their influence. Bivariate analyses revealed some significant relationships between the IDEAS model variables and gender, and with car ownership (a limited proxy for socioeconomic status) but we found none involving religion or other SES indicator variables. In order to assess potential confounds with gender and car ownership, *Model 2d* included these variables with significant correlations as additional covariates in the model already specified by Model 2c. This did not substantively affect the model fit or any of the significant pathways and no additional pathways were suggested by modification indices.

Supplementary Table S1. Model fit statistics for different variants of the model tests.

| Model |  | RMSEA | SRMR | CFI | χ^2^ | df |
| --- | --- | --- | --- | --- | --- | --- |
| 1* | Basic IDEAS | .059 | .064 | .939 | 1175.837 | 259 |
| 1b** | Add political interest to intention | .062 | .067 | .929 | 1271.32 | 260 |
| 1c | Add age covariate | .062 | .067 | .924 | 1359.912 | 279 |
| 2a | As Model 1 but treat prospective efficacy (items 2,3) as efficacy with current efficacy as covariate | .075 | .056 | .941 | 1088.962 | 254 |
| 2b | As Model 1 but treat current efficacy (items 1) as efficacy with prospective efficacy as covariate | .065 | .069 | 921 | 1361.77 | 256 |
| 2c | As Model 1 but treat prospective efficacy (items 2,3) as efficacy dropping current efficacy item | .056 | .045 | 945 | 980.205 | 232 |
| 2d | As Model 2c but path from political interest to vote intention, and adding gender, car ownership (SES), and age as covariates. | .056 | .050 | .936 | 1196.548 | 288 |
| 3 | Add to Model 2d the paths specified as non-significant – from PRD to social change beliefs and to vote intention. | .052 | .047 | .941 | 1181.294 | 313 |

Note: Based on N = 1012; *retaining significant covariances between CogCRD, and both PRD and Identification;

** otherwise as Model 1. Chi Square is significant for all models (*p* < .001)

Figure 3. SEM test of the Hypothesized pathways in the Identity-Deprivation-Efficacy-Action-Subjective Well-Being (IDEAS) model, showing results for Model 3, with age, gender and car ownership as covariates. RMSEA = .052, SRMR = .047, CFI = .941, χ^2^ (313) = 1181.294, p <.001. Pathways theoretically specified to be non-significant are shown with dotted lines. H = hypothesis test: H1 Ideology; H2 Identification; H3 Collective Deprivation; H4 Indirect Identification via Discrimination; H5 Collective Efficacy; H6 Personal Deprivation; H7 Bolster; H8 Affective Crossover. All coefficients are standardized and are significant at *p* <. 001, except * *p* < .05 and ** *p* < .01.

008. ns

*H8: -*.201

*H5a* .148

*H8: -*.192

*H7:* .115

*H3b* .463

*H3b* .436

Vote for

Independence

*R*^2^ = .82

PersonalRD

*H3d* .237

*H5c:* .107

*H1:* .906

*H6 -*.484

CognitiveCollective RD

*H4* .188

*H3a .*266

*H5b* .083*

*H3c* .410

*H2:* .196

*r* = .251

-0.013, *ns*

*r* = .307

Political

Engagement

.018, *ns*

034. ns

**Indirect Effects**

From Model 2c we also tested specific indirect effects using PROCESS V3 Model 6. Further details are available on request.

Supplementary Table S2. *Indirect Effects on Social Change Beliefs and Subjective Well Being.*

| Indirect Effect Tested | Standardized  Effect | *SE* | *Z* | *p* |
| --- | --- | --- | --- | --- |
| a) Identification > Efficacy > Social Change Beliefs | .012 | .007 | 2.14 | .032 |
| b) Identification > Discrimination > Social Change Beliefs | .069 | .02 | 4.42 | < .001 |
| c) Identification > Discrimination > Efficacy > Social Change Beliefs | .001 | .001 | 1.79 | .074 |
|  |  |  |  |  |
| a) Identification > Discrimination > Well-being | -.02 | .007 | 2.45 | .014 |
| b) Identification > Emotions > Well-being | .005*ns* | .005 | 0.81 | .42 |
| c) Identification > Discrimination > Negative Emotions > Wellbeing | -.017, | .004 | 3.40 | .001 |

Note: CogCRD is allowed to covary with identification and PRD as these are exogenous variables

Table S2 shows that there are significant indirect effects of identification on social change beliefs via higher efficacy and greater perceived discrimination, separately but not sequentially. This is in addition to the direct effect of identification on social change beliefs. Table S2 also shows that there is a significant indirect *negative* effect of identification on well-being through perceived discrimination, and through perceived discrimination and then negative emotions, but not solely through negative emotions. This is in addition to the significant positive direct effect of identification on well-being.

Additional indirect tests were requested by an anonymous reviewer. These are summarised in Supplementary Table S3 below.

Supplementary Table S3. *Indirect Effects on Voting Intention via Social Change Beliefs.*

| Indirect Effect Tested | Standardized  Effect | *SE* | *Z* | *p* |
| --- | --- | --- | --- | --- |
| Identification > Social Change Beliefs > Vote Intent | .157 | .012 | 6.36 | < .001 |
| Identification > Discrimination > Social Change Beliefs > Vote Intent | .062 | .007 | 4.42 | .001 |
| Identification >Efficacy > Social Change Beliefs > Vote Intent | .011 | .002 | 2.15 | .005 |
| Identification > Discrimination > Efficacy > Social Change Beliefs > Vote Intent | .001 | .000 | 1.79 | .074 |
| Identification >Discrimination > Emotions > Social Change Beliefs > Vote Intent | .017 | .002 | 3.49 | < .001 |

There is no direct effect of identification on voting intention, (.017, *SE* = .008, *Z* = 1.01, *p* = .311). Table S3 confirms that identification has significant indirect effects on voting intention, primarily via social change beliefs, but also via effects on discrimination, efficacy, and discrimination, then emotions, which then affect social change beliefs.

Reviewers also asked us to consider alternative models including SIMCA, the Rejection Identification model and the Elaborated Social Identity Model. We wish to emphasise that the following additional analyses were not specified by our model and therefore they represent post-hoc considerations.

SIMCA holds that identification should predict discrimination, emotion and efficacy should directly predict voting. However, our model already shows that these paths are all mediated through social change beliefs. The rejection identification model holds that perceived discrimination should predict identification, which should then predict well-being. We are aware of other evidence that shows the directionality of the paths between identification and perceived discrimination may be reciprocal or reversed at times. We tested this model versus our own (identification predicts perceived discrimination, which affects well-being) and found that the RI model is less well supported. Specifically, the indirect effect of discrimination on well-being via identification is non-significant (.009, *SE* = .01, *Z*  = .546, *p* = .585), whereas the indirect effect of identification on well-being via discrimination is highly significant (-.058, *SE* = .01, *Z*  = -4.926, *p* < .001), Nonetheless, both approaches hold that both identification and discrimination should bear on well-being.

We were also asked to consider how system justification theory might relate to our model. The theory assumes that system justification (which we will interpret as relating to the inverse of social change beliefs) predicts other psychological measures which then predict collective action. In Jost et al. (2017) a series of different variants of system justification theory predictions are offered, though these related to normative and non-normative collective action, not voting intentions. These models are as follows: a) social change beliefs should predict voting intention via emotions, b) social change beliefs, emotions and perceived discrimination should simultaneously and separately predict voting intention, c) social change beliefs should predict identification , which should predict voting, and d) efficacy, identification and social change beliefs should all simultaneously and separately predict voting intention. Because our model and data show that any effects of identification, emotions, efficacy and perceived discrimination flow through social change beliefs, we conclude that none of the system justification models set out in the Jost et al. (2017) paper fit the data. For example, a simple model of identification affecting voting intention via social change beliefs fit the data better (CFI = .948, RMSEA = .057, Chi square (235df) = 1008.051) than does a simple model of social change beliefs affecting voting intention via identification (CFI = .856, RMSEA = .095, Chi square (235df) = 2382.72).

The Elaborated Social Identity Model (Drury & Reicher, 1999) concerns the exhilarating effects of engaging in crowd action, whereby the strengthening of social identity also gives rise to positive emotions and hence may affect well-being. Although we find this a very persuasive model, it is not very relevant to the situation of individuals planning their intended vote in a private polling booth. Thus, although there are certainly reasonable conceptual parallels, the ESIM does not directly bear on the predictions for the present study.
